# Rates of Prevalent HIV Infection, Prevalent Diagnoses, and New Diagnoses Among Men Who Have Sex With Men in US States, Metropolitan Statistical Areas, and Counties, 2012-2013

**DOI:** 10.2196/publichealth.5684

**Published:** 2016-05-17

**Authors:** Eli Samuel Rosenberg, Jeremy Alexander Grey, Travis Howard Sanchez, Patrick Sean Sullivan

**Affiliations:** ^1^ Department of Epidemiology Rollins School of Public Health Emory University Atlanta, GA United States

**Keywords:** HIV surveillance, HIV diagnosis, HIV prevalence, HIV incidence, men who have sex with men, gay and bisexual men, demography

## Abstract

**Background:**

In the United States, men who have sex with men (MSM) increasingly represent the majority of people living with and acquiring human immunodeficiency virus (HIV) infection. Local and federal surveillance programs estimate the number of persons living with an HIV diagnosis, persons living with HIV infection, and new diagnoses. Given the absence of population-based estimates of the number of MSM for US states, metropolitan statistical areas (MSAs), or counties, it is not possible to accurately estimate rates using these indicators at these levels, inhibiting the ability to understand HIV burden and to direct prevention efforts.

**Objective:**

To synthesize recently published estimates of MSM population size with publicly available HIV surveillance data, in order to estimate the prevalence of HIV diagnosis and infection and the rate of new diagnoses, at the national, state, MSA, and county levels.

**Methods:**

The number of MSM living with HIV infection in 2012 (prevalence), living with an HIV diagnosis in 2012 (diagnosed prevalence), and newly diagnosed with HIV infection in 2013 (new diagnosis), at state, MSA, and county levels, were obtained from publicly available data from AIDSVu.org and the US Centers for Disease Control and Prevention. The estimated number of MSM living in every US county was calculated using recently published methodology that utilized data from the National Health and Nutrition Examination Survey and American Community Survey. Estimated county-level MSM counts were aggregated to form MSA- and state-level totals. From this, we estimated HIV prevalence, diagnosed prevalence, and new diagnosis rates.

**Results:**

The estimated HIV prevalence among MSM in the United States in 2012 was 15.0% (666,900/4,452,772), the diagnosed HIV prevalence in 2012 was 11.1% (493,453/4,452,772), and the new diagnosis rate for 2013 was 0.7 per 100 MSM. For diagnosed prevalence at the state level, 6 states had both <15,000 cases and diagnosed prevalence rates of ≥15%, all in the South. Five highly populated states had ≥15,000 cases and rates between 10% and 15%. Georgia was the only state with ≥15,000 cases and ≥15% diagnosed prevalence rate. Of the 25 MSAs with the highest diagnosed prevalence rates in the United States, 21 were in the South and 6 had diagnosed prevalence of ≥25%. County-level data showed high diagnosed prevalence rates in both urban and rural counties of the South.

**Conclusions:**

HIV infection is hyperendemic among MSM in many areas of the United States, particularly in the South. Our data emphasize the priorities for HIV prevention and care set forth in the United States National HIV/AIDS Strategy (NHAS) and provide updatable local estimates of NHAS indicators. Jurisdictions can use these results to direct resources, programs, and policies to optimally benefit the health of MSM.

## Introduction

The United States’ human immunodeficiency virus (HIV) epidemic has long been characterized by a concentration of infection among men who have sex with men (MSM). MSM accounted for an estimated 53% of people living with a diagnosis of HIV in 2013 and 67% of new HIV diagnoses in 2014, despite representing a minority of men in the United States [1, 2]. Furthermore, individuals of color and those living in the Southern United States also comprise a disproportionate share of new infections, new diagnoses, those living with an HIV diagnosis, and deaths among persons living with an HIV diagnosis [1, 3, 4]. The intersection of these groups, black MSM in the South, represents the most affected subgroup in the United States, for whom extremely high levels of HIV prevalence and incidence have been recorded across a variety of surveillance and research designs [5-9]. The 2015 update to the United States National HIV/AIDS Strategy (NHAS) places priority on MSM, particularly black MSM, and individuals living in the South, and establishes indicators to monitor progress in reducing the extent of HIV disparities in these populations, to monitor the state of the US epidemic, and to guide the allocation of prevention efforts [10]. The implementation of the NHAS will require intensive local action, informed by local estimates of the extent of HIV infection [11].

To best understand the burden of existing and new HIV infections among MSM at subnational levels, high-quality data sources for both infection numerators and population-size denominators are needed, yet historically have been incomplete. In the United States, HIV infection is reportable; since 2004, name-based diagnoses of HIV infection have been reported by all 50 states to the Centers for Disease Control and Prevention (CDC). CDC routinely releases HIV surveillance data in reports and on HIV Atlas [1, 5, 12]. These data include estimated state-, county-, and metropolitan statistical area (MSA)-level counts of MSM newly diagnosed with HIV infection in each year and all MSM currently living with diagnosed HIV infection. AIDSVu.org is a website that uses these data to develop highly detailed maps and other visualizations of the HIV epidemic and allows downloads of county-specific CDC data on HIV prevalence [13]. There is no direct surveillance measurement of the total number of MSM living with HIV infection (ie, including those not yet diagnosed), but CDC has used existing surveillance data to model the number of MSM living with HIV infection at the state level [14].

Unlike for other groupings of persons (eg, by race, sex, or age) for which CDC publishes both counts and rates of HIV diagnoses and prevalence, surveillance-based estimates for MSM are available only as counts. This limits our understanding of not only the total burden of HIV infection among MSM but also the relative burden across geographic areas in which numbers of MSM may vary. CDC led an effort to create a national estimated rate of the MSM living with diagnosed HIV infection using surveillance data from 37 states and an MSM population-size denominator based on a synthesis of data sources, primarily the National Health and Nutrition Examination Survey (NHANES) [2]. CDC also funds an ongoing supplemental surveillance project in 20 of the largest US cities that directly measures the rate of prevalent HIV infection in a nonrepresentative sample of MSM [6-8, 15, 16]. These data are valuable for understanding the impact of HIV infection among MSM in those cities, but other areas need similar estimates to better understand their own community’s HIV epidemic and to target resources appropriately. This is particularly critical as part of the nationwide call for organizations to incorporate the goals and indicators from the NHAS into their program plans [10, 11]. The rate of new HIV diagnoses is a central indicator for success in the NHAS goals of reducing new HIV infections (Goal 1, Indicator 2) and reducing HIV-related health disparities for MSM (Goal 4, Indicator 9) [10].

To generate these rates at the state, county, and MSA levels, we needed new data on the number of MSM in each of those areas. We have recently published a method that allocates the national proportion of MSM to all US states, counties, and MSAs, using additional NHANES results and American Community Survey (ACS) data [17, 18]. Here, we apply these new denominators to the publicly available HIV case surveillance data to obtain rates of HIV diagnoses and prevalence among MSM at national, state, county, and MSA levels.

## Methods

We estimated and examined a variety of HIV infection indicators for MSM at multiple levels by combining publicly available disease numerator data with denominators for MSM, as follows.

### Numerator Data Sources and Methods

Estimated MSM diagnosis and infection count data were extracted from public CDC and AIDSVu.org data sources to inform estimates at the national level and all 50 US states plus the District of Columbia (Table 1) [1, 12, 13]. County-level data on prevalence of persons living with diagnosed HIV infection (diagnosed prevalence) among MSM came from AIDSVu.org [13]. MSA-level data on diagnosed HIV prevalence and new HIV diagnoses among MSM came from a CDC surveillance report [5]. 

To protect the privacy of persons living with HIV infection in smaller communities, standard publication practices for HIV surveillance data suppress county data where there are fewer than 5 cases; this includes stratified counts, such as for MSM. In addition, CDC adheres to agreements with each individual state regarding additional restrictions on the public release of HIV surveillance data at the county level. Although for the 2012-2013 HIV case surveillance data most states (n=32) allowed CDC to release data about MSM from all counties that met the 5+ case rule, many either only allowed release of these data from large mostly urban counties (n=10) or did not allow the data to be released at all (n=8).

**Table 1:** HIV burden indicators and sources of numerator HIV data.

Level	Prevalence of living with an HIV^a^ diagnosis, 2012	Rate of new HIV diagnoses, per MSM^b^, 2013	Rate of new HIV diagnoses, per MSM without an HIV diagnosis, 2013	Prevalence of HIV infection, 2012	Prevalence of undiagnosed HIV infection, 2012
National	AIDSVu [13]	AIDSVu	AIDSVu	MMWR^c^ [14]	MMWR
State	AIDSVu	AIDSVu	AIDSVu	MMWR	MMWR
MSA^d^	CDC^e^Surveillance Report [5]	CDC Surveillance Report	CDC Surveillance Report	—	—
County	AIDSVu	—	—	—	—

^a^HIV: human immunodeficiency virus.

^b^MSM: men who have sex with men.

^c^MMWR: Morbidity and Mortality Weekly Report.

^d^MSA: metropolitan statistical area.

^e^CDC: Centers for Disease Control and Prevention.

### Denominator Data Sources and Methods

We used the method by Grey et al [17] to estimate the number of MSM in the United States in 2012 and 2013. In brief, we began with 2 published estimates. The first was the result of a meta-analysis-based estimate of the percentage of US men who have had sex with another man in the past 5 years [2]. The second, from an analysis of NHANES data, was the percentages of US men who had sex with another man in the past year, at each of 4 levels of urbanicity: large central metropolitan areas, large fringe metropolitan areas, medium or small metropolitan areas, and nonmetropolitan areas [18]. We next evaluated the percentage of same-sex male couple households among all households in each county, from the ACS [19]. We then calculated the ratio of these percentages to the percentage among all counties at the same level of urbanicity. By multiplying these ratios by the percentages reported by Oster et al [18], we assigned each county a new, locally tailored estimated percentage of men who had sex with a man in the past year among adult men, a method similar to that published by Lieb et al [20]. Finally, we multiplied each county’s estimate by the number of adult men in the county, according to the ACS. In order to estimate the number of men who had sex with a man in the past 5 years, generally considered more indicative of the sexually active MSM population, we scaled the single-year population sizes to equal the estimate given by Purcell et al [2] of 3.9% of the US adult male population. To obtain estimates at the state and MSA levels, we aggregated MSM population size estimates from their composite counties.

In addition to the numerator-based suppression previously mentioned, standard publication practices for HIV surveillance data also suppress the display of county-level rates where there are fewer than 100 persons in the population, including in any single group stratum, such as MSM. Ultimately, of the 1521 counties with available and unsuppressed data on MSM living with an HIV diagnosis, an additional 253 (16%) had fewer than 100 MSM according to our estimation method. The remaining 1268 counties, which contained 84% of the US adult male population and 89% of all MSM living with an HIV diagnosis in 2012, contributed to the county-level analysis.

### HIV Burden Indicators

At the state level, we computed the rates of HIV prevalence in 2012 (diagnosed and undiagnosed), diagnosed prevalence in 2012, and new diagnoses in 2013. Prevalence rates were among all MSM; new diagnosis rates were among all MSM and among only MSM not previously diagnosed with HIV (total MSM in 2013 minus total MSM living with a diagnosis at year-end of 2012). State-level estimates were aggregated to yield national estimates. At the county level, we computed the rates of diagnosed HIV prevalence among all MSM in 2012. At the MSA level, we computed the rates of diagnosed HIV prevalence in 2012 and new diagnosis in 2013 among all MSM.

### Sensitivity Analyses

To explore the potential effect of regional underreporting or within-urbanicity stratum underrepresentation of male-male cohabitation on the ACS-based Grey estimator, we also produced HIV prevalence and new diagnosis rates using MSM denominators according to more simplified models that assumed estimates from Oster et al and Purcell et al (see Supplement for details) [2].

## Results

**Figure 1:**
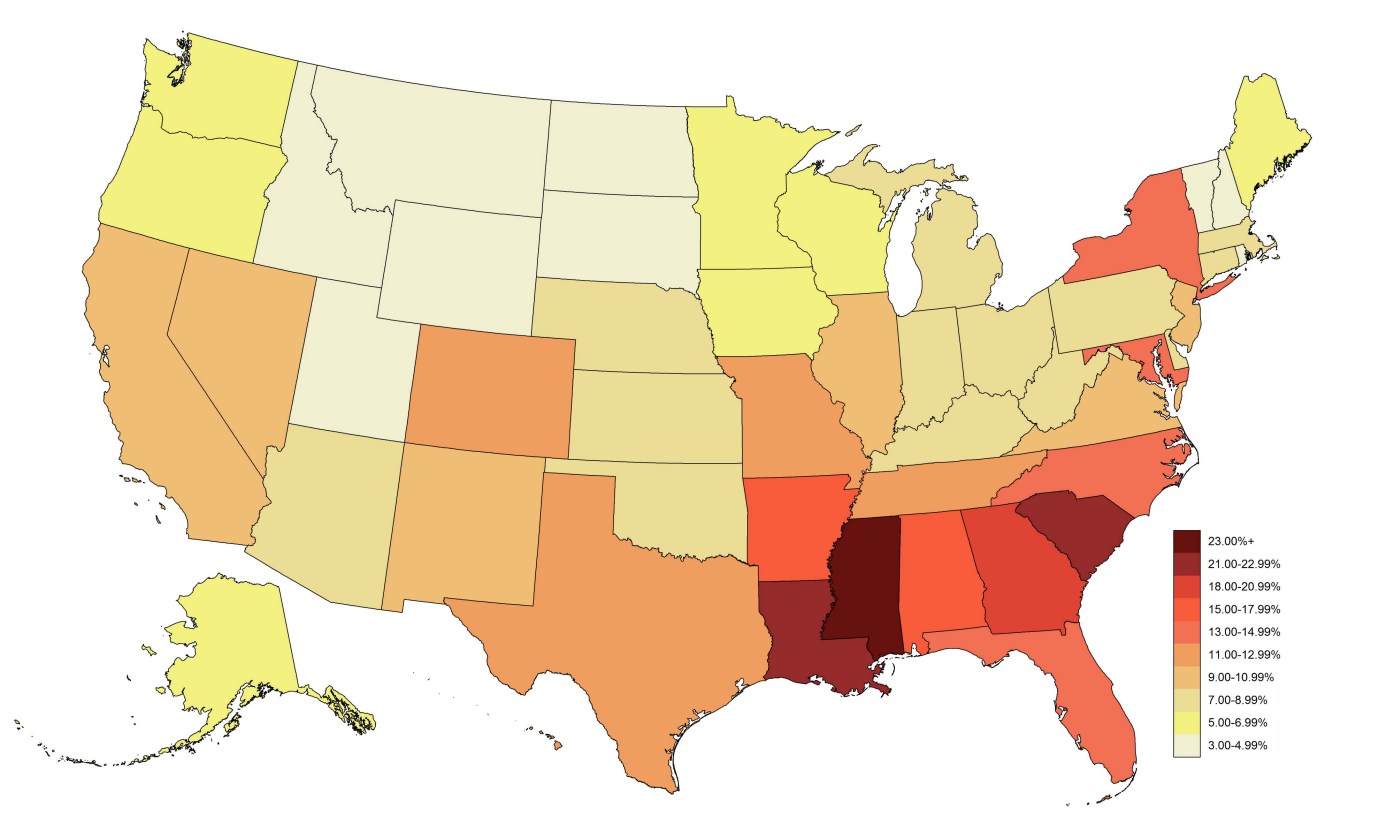
Prevalence of human immunodeficiency virus diagnoses among men who have sex with men (MSM) per 100 MSM, by US states and District of Columbia, 2012.

**Figure 2:**
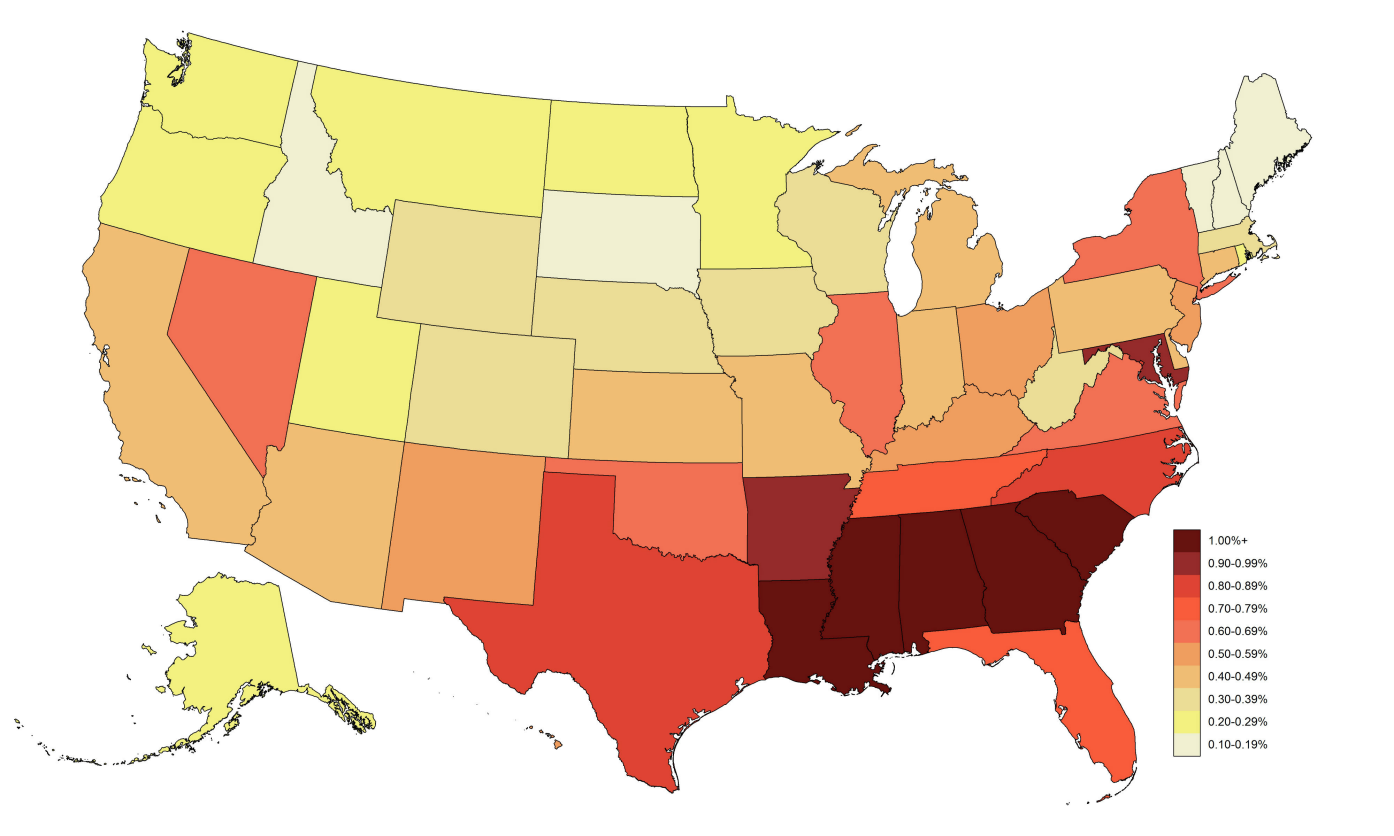
Rate of new human immunodeficiency virus diagnoses among men who have sex with men (MSM) per 100 MSM, by US states and District of Columbia, 2012-2013.

In the United States in 2012, an estimated 15.0% of MSM were living with HIV infection (diagnosed and undiagnosed) and 11.1% were living with an HIV diagnosis (Tables 2-3, Figure 1). Using the comparison method in Purcell et al [2], the diagnosed HIV prevalence rate among MSM was 57.5 times greater than among other US men. States in the South had the highest rates of diagnosed HIV prevalence among MSM, with all rates of ≥15% located in the South (Table 2, Figure 1). Among states, the rates of diagnosed HIV prevalence among MSM living in Louisiana, Mississippi, and South Carolina were all approximately twice the national rate. The rates of new HIV diagnoses among MSM for 2013 at the state level strongly followed the same pattern as HIV prevalence (Table 2, Figure 2). Only southern states had new diagnosis rates per MSM and per MSM without an HIV diagnosis of ≥1.00/100 MSM, with 2 (Louisiana and Mississippi) having rates per MSM without an HIV diagnosis of ≥2.00/100 MSM.

**Figure 3:**
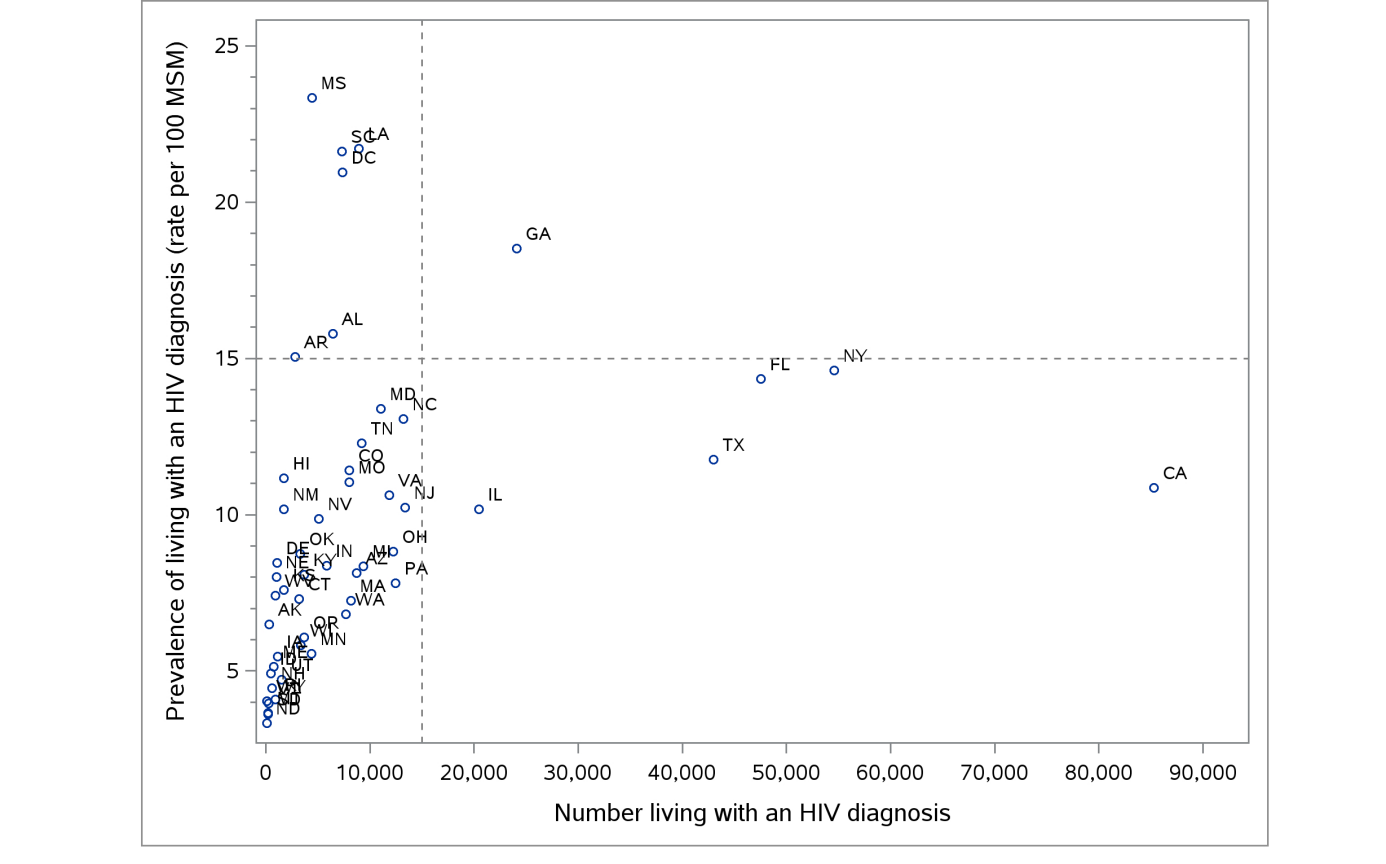
Prevalence versus number of men who have sex with men (MSM) living with a human immunodeficiency virus (HIV) diagnosis, by US states and District of Columbia, 2012.

Plotting the diagnosed HIV prevalence case counts versus rates among MSM in 2012 (Figure 3), 4 groups are notable: populous states with ≥15,000 cases and diagnosed prevalence between 10% and 15% (California, Florida, Illinois, New York, Texas), southern states with diagnosed prevalence of ≥15% and <15,000 cases, and states with diagnosed prevalence of <15% and <15,000 cases. Georgia is uniquely high in both the rate and case count of MSM living with diagnosed HIV infection.

**Figure 4:**
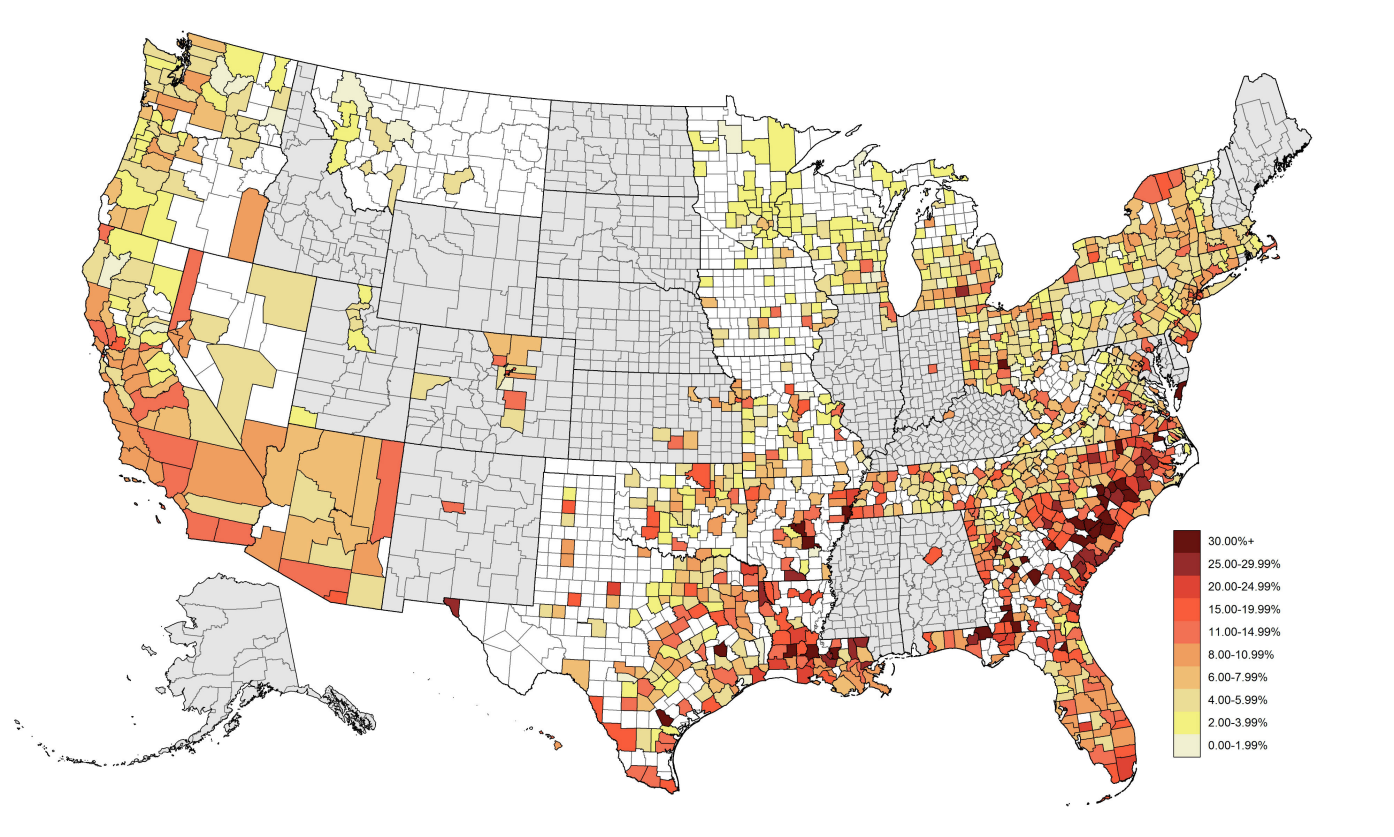
Prevalence of human immunodeficiency virus (HIV) diagnoses among men who have sex with men (MSM) per 100 MSM, by US counties, 2012. Counties in gray represent areas for which data were not permitted to be released by the state. Those in white represent areas for which data were available, but either the numerator or denominator was suppressed. The remaining counties with available and unsuppressed data represented 84% of US adult males and 89% of prevalent US MSM HIV diagnoses. A data file with the county-level HIV prevalence rate data is available on the Emory CAMP website [37]. Because all US counties are not included, it is inadvisable to rank counties based on HIV burden.

All but 4 high-prevalence MSAs are located in the South, and 6 southern MSAs have diagnosed HIV prevalence rates among MSM for 2013 of ≥25% (Table 4, complete MSA data provided in supplement). The map of county-level diagnosed HIV prevalence rates among MSM in 2012 shows a similar pattern of high-prevalence urban areas (Figure 4). This map also shows that several rural counties in the South have diagnosed HIV prevalence rates of ≥20% and ≥30% (Figure 4).

The new MSM population size estimation by Grey et al also appears to produce similar state-level HIV rates to previous approaches and, in the instances of certain MSAs, more plausible results (see Supplement for full sensitivity analysis results).

**Table 2:** Prevalence of HIV diagnoses and rates of new diagnoses among men who have sex with men, by US states and District of Columbia, 2012-2013.

State	MSM^a^ living with an HIV^b^ diagnosis, 2012		New MSM HIV diagnoses, 2013		
	n	Rate per 100 MSM	n	Rate per 100 MSM	Rate per 100 MSM without an HIV diagnosis
Alabama	6442	15.78	442	1.09	1.29
Alaska	335	6.49	12	0.24	0.25
Arizona	8748	8.14	513	0.46	0.50
Arkansas	2843	15.04	192	1.00	1.17
California	85,307	10.85	3860	0.49	0.55
Colorado	8028	11.42	241	0.33	0.37
Connecticut	3178	7.30	188	0.43	0.47
Delaware	1115	8.45	60	0.46	0.50
District of Columbia	7360	20.96	313	0.85	1.06
Florida	47,520	14.35	2711	0.80	0.93
Georgia	24,101	18.51	1708	1.30	1.59
Hawaii	1758	11.16	78	0.51	0.57
Idaho	477	4.92	15	0.15	0.16
Illinois	20,495	10.17	1273	0.64	0.71
Indiana	5876	8.37	332	0.47	0.52
Iowa	1133	5.45	77	0.37	0.39
Kansas	1723	7.59	109	0.48	0.51
Kentucky	3697	8.08	260	0.55	0.60
Louisiana	8954	21.72	730	1.76	2.24
Maine	771	5.13	21	0.14	0.15
Maryland	11,052	13.38	762	0.90	1.04
Massachusetts	8181	7.24	443	0.40	0.43
Michigan	9377	8.34	547	0.48	0.52
Minnesota	4416	5.54	202	0.24	0.26
Mississippi	4469	23.34	316	1.66	2.18
Missouri	7994	11.04	341	0.48	0.54
Montana	239	3.66	19	0.30	0.31
Nebraska	1015	8.01	51	0.39	0.42
Nevada	5070	9.86	329	0.64	0.71
New Hampshire	621	4.44	21	0.15	0.16
New Jersey	13,402	10.23	790	0.60	0.66
New Mexico	1729	10.18	102	0.57	0.63
New York	54,606	14.61	2264	0.61	0.72
North Carolina	13,202	13.06	859	0.83	0.96
North Dakota	132	3.33	13	0.29	0.30
Ohio	12,259	8.81	767	0.53	0.58
Oklahoma	3293	8.74	236	0.63	0.69
Oregon	3673	6.07	159	0.26	0.27
Pennsylvania	12,477	7.81	739	0.45	0.49
Rhode Island	949	4.09	54	0.23	0.24
South Carolina	7332	21.63	452	1.24	1.56
South Dakota	196	3.62	9	0.17	0.18
Tennessee	9198	12.29	563	0.76	0.87
Texas	42,973	11.77	3129	0.84	0.95
Utah	1532	4.71	72	0.22	0.23
Vermont	275	3.96	12	0.17	0.18
Virginia	11,888	10.63	683	0.61	0.68
Washington	7681	6.82	325	0.29	0.31
West Virginia	930	7.41	46	0.35	0.38
Wisconsin	3388	5.82	190	0.32	0.34
Wyoming	133	4.04	11	0.34	0.36
*50 US states & Washington, DC*	*493,453*	*11.08*	*27,641*	*0.61*	*0.69*

^a^MSM: men who have sex with men.

^b^HIV: human immunodeficiency virus.

**Table 3:** Prevalence of HIV infection and undiagnosed HIV infection among men who have sex with men, by US states, 2012.

State	MSM^a^ living with HIV^b^ infection, 2012		MSM living with undiagnosed HIV infection, 2012	
	n^c^	Rate per 100 MSM	n^c^	Rate per 100 MSM
Alabama	7900	19.36	1600	3.92
Alaska^d^	410	7.94	20	0.39
Arizona	10,500	9.77	1200	1.12
Arkansas	3500	18.51	800	4.23
California	134,400	17.10	16,400	2.09
Colorado	8900	12.66	950	1.35
Connecticut	4600	10.57	710	1.63
Delaware	1600	12.13	240	1.82
District of Columbia	11,300	32.18	1400	3.99
Florida	60,500	18.27	8100	2.45
Georgia	33,100	25.42	6900	5.30
Hawaii	2500	15.87	220	1.40
Idaho^d^	630	6.50	80	0.83
Illinois	27,800	13.79	5300	2.63
Indiana	6900	9.83	1000	1.42
Iowa	1600	7.70	330	1.59
Kansas	2200	9.70	380	1.67
Kentucky	5300	11.59	890	1.95
Louisiana	10,700	25.96	2700	6.55
Maine^d^	1200	7.99	90	0.60
Maryland	16,200	19.61	3900	4.72
Massachusetts	12,200	10.80	2000	1.77
Michigan	10,900	9.69	1900	1.69
Minnesota	5200	6.53	770	0.97
Mississippi	5400	28.20	1200	6.27
Missouri	9100	12.57	1500	2.07
Montana^d^	420	6.43	30	0.46
Nebraska^d^	1300	10.25	190	1.50
Nevada	6500	12.64	1000	1.94
New Hampshire^d^	950	6.79	120	0.86
New Jersey	16,800	12.83	3700	2.83
New Mexico	2400	14.13	280	1.65
New York	75,900	20.30	7700	2.06
North Carolina	16,100	15.93	2600	2.57
North Dakota^d^	190	4.79	20	0.50
Ohio	14,800	10.63	3100	2.23
Oklahoma	4100	10.88	740	1.96
Oregon	5800	9.58	850	1.40
Pennsylvania	16,100	10.08	2700	1.69
Rhode Island	1100	4.75	200	0.86
South Carolina	9500	28.03	2000	5.90
South Dakota	200	3.70	30	0.55
Tennessee	11,000	14.70	1800	2.40
Texas	62,400	17.09	12,100	3.31
Utah	1700	5.23	250	0.77
Vermont^d^	520	7.49	0	0
Virginia	13,500	12.07	2000	1.79
Washington	10,400	9.23	1300	1.15
West Virginia^d^	1200	9.56	200	1.59
Wisconsin	4000	6.87	650	1.12
Wyoming^d^	180	5.47	40	1.22
*50 US states & Washington, DC*^e^	*666,900*	*14.98*	*98,700*	*2.22*

^a^MSM: men who have sex with men.

^b^HIV: human immunodeficiency virus.

^c^Counts are rounded model-based estimates, per the source Centers for Disease Control and Prevention (CDC) report [14].

^d^Counts indicated as numerically unstable, per the source CDC report [14].

^e^Total counts calculated by different methodology than used for jurisdictions and thus do not sum to column totals, per the source CDC report [14].

**Table 4:** Prevalence of HIV diagnoses and rates of new diagnoses among men who have sex with men, by top 25 HIV prevalence rates among US metropolitan statistical areas, 2012-2013.

Metropolitan statistical area^a^	MSM^b^ living with an HIV^c^ diagnosis (2012)		HIV diagnoses among MSM (2013)		
	n	Rate per 100 MSM	n	Rate per 100 MSM	Rate per 100 MSM without an HIV diagnosis
Atlanta–Sandy Springs–Roswell, GA	12,532	16.43	1393	1.36	1.62
Augusta–Richmond County, GA–SC	691	26.56	89	2.79	3.91
Baton Rouge, LA	1087	25.40	155	2.91	4.14
Birmingham–Hoover, AL	1809	13.35	130	0.79	0.92
Charleston–North Charleston, SC	931	20.08	84	1.46	1.81
Chattanooga, TN–GA	577	15.06	35	0.85	1.02
Columbia, SC	1619	29.65	99	1.43	1.96
Dayton, OH	807	13.84	61	0.89	1.02
Durham–Chapel Hill, NC	741	17.47	70	1.13	1.34
El Paso, TX	1131	28.53	96	2.22	3.11
Fresno, CA	998	13.79	88	1.16	1.35
Greensboro–High Point, NC	975	20.98	75	1.26	1.58
Greenville–Anderson–Mauldin, SC	806	16.07	80	1.42	1.69
Jackson, MS	1201	39.49	106	2.51	4.05
Little Rock–North Little Rock–Conway, AR	1081	23.33	124	2.24	2.87
Los Angeles–Long Beach–Anaheim, CA	34,919	12.92	1938	0.62	0.71
McAllen–Edinburg–Mission, TX	494	14.12	66	1.60	1.88
Memphis, TN–MS–AR	2954	17.61	257	1.18	1.43
Miami–Fort Lauderdale–West Palm Beach, FL	21,482	17.52	1592	1.13	1.35
New Orleans–Metairie, LA	3091	19.71	356	1.68	2.08
New York–Newark–Jersey City, NY–NJ–PA	46,869	14.61	3007	0.76	0.89
Tulsa, OK	944	14.08	111	1.47	1.70
Virginia Beach–Norfolk–Newport News, VA–NC	2930	14.40	242	0.97	1.13
Washington–Arlington–Alexandria, DC–VA–MD–WV	12,606	13.43	1105	0.90	1.03
Winston-Salem, NC	606	16.08	54	1.11	1.33

^a^Metropolitan statistical area (MSA) results are provided alphabetically for the top 25 MSAs (based on diagnosed prevalence rates). The results for the remaining MSAs are available in the supplement.

^b^MSM: men who have sex with men.

^c^HIV: human immunodeficiency virus.

## Discussion

Despite incredible achievements in prevention, HIV diagnosis, and antiretroviral therapies over the 35 years of the epidemic, the impact of HIV in US communities of MSM remains staggering. We examined prevalence of HIV at the state and county levels, and found that, although there are important local variations in HIV prevalence, the prevalence of HIV among MSM is consistently orders of magnitude higher than among other Americans. Whereas prevalence of HIV is routinely expressed in cases per 100,000 persons for other groups, for MSM, we report cases per 100 persons [5]. The levels of prevalence among MSM correspond to international benchmarks for epidemicity or hyperendemicity, with all 50 states at least tripling the prevalence criterion for generalized epidemics among MSM (>1% prevalence) and 6 states (all in the South) meeting the UNAIDS criterion for hyperendemicity (>15% prevalence) [21]. The county-level findings demonstrate that although most HIV infections among MSM are concentrated among urban-dwelling MSM, rural areas still represent important places of HIV burden that may be underserved by HIV prevention and care services [5, 22]. Furthermore, rates of new diagnoses among MSM provide insight into the ongoing trajectory of the epidemic, providing a national, regional, and local view of an epidemic force of infection that requires urgent response and prioritization [23].

Our analysis builds on previous reports of the importance of local variations in social and structural epidemic determinants [24], and underscores previous identification of the US South as the area of greatest concentration of HIV infection [10]. In terms of state-specific HIV prevalence rates among MSM, 9 of the top 10 are in the South. We also have new evidence that the HIV epidemic in the South is continuing to grow disproportionately to other regions. New HIV diagnoses rates among MSM for 2013 show that the top 13 states were all in the South, with 10 states having new diagnoses rates of >1% per year. These concentrations of new diagnoses in the South might represent relative increases in new transmissions or could represent previous successes in reducing HIV transmissions among MSM outside the South [25-27]. Our analysis furthers previous surveillance reports and analyses by recognizing that there are differences in the distribution of MSM by state and by county; having high-quality estimates of MSM denominators allows for more reliable comparisons in prevalence and diagnosis rates across geographic areas. Denominators also allow for a more direct quantification of the health disparity among MSM than do rates expressed against a denominator of all men.

Our estimates of HIV prevalence differ importantly from previous estimates of HIV prevalence in the United States. The study by Purcell et al, based on data from 37 states, reported a diagnosed HIV prevalence among MSM in 2007 of about 8% [2, 28]. Our estimate for 2012, including data from all states and the District of Columbia, is that diagnosed HIV prevalence was 11% and that, including estimated undiagnosed infections, 15% of US MSM live with HIV infection. Previous data from the National HIV Behavioral Surveillance System (NHBS) have shown higher HIV prevalence (18% in 2011) among MSM recruited in high-risk venues in the 20 largest cities in the United States [29, 30]; these differences likely reflect selection bias for men in high-risk venues, and the higher risk of HIV infection in urban areas. Our findings have relative advantages over NHBS-derived estimates because of population-based sources of numerator and denominator data and inclusion of areas outside the largest US cities, including rural areas.

Evaluating and monitoring data in smaller geographic areas is critical to making effective local responses and to measuring their impact. Indeed, the first step of the first goal of the NHAS is to assess the communities in which HIV is more *concentrated*[11]. Our data are also of relevance to the updated National Strategy [31]. In terms of Goal 3, reducing HIV-related disparities, our new data illustrate the extent of the health disparity among MSM—a staggering 57-fold disparity in diagnosed HIV prevalence rates among MSM compared with other US men. Because our method of calculating rates of new HIV diagnoses among MSM can be updated annually, it can also serve as a source of data to evaluate Indicator 9, measures of health disparities among MSM. The updated NHAS uses a denominator of all men to calculate the rate of new HIV diagnoses among MSM nationally, which was the best available data at the time. However, at the local level, similar metrics will have varying amounts of bias, depending on the relative concentration of MSM in a particular jurisdiction. Thus, for local planning and evaluation, rates based on local MSM denominators should be used. To most comprehensively address this purpose, it will require developing similar race-specific estimates of MSM at the state and county levels. Such data are not yet available but would be important for the ongoing evaluation of an epidemic with profound disparities by race and ethnicity.

### Limitations

These results have important limitations. Because all residential locations informing HIV case numerators were determined at the time of diagnosis, and the denominators were based on recent ACS data, postdiagnosis migration and undercounting of nonresidents may contribute to mismeasurement. Furthermore, there are differences in the time frames for assessment of male-male sex for numerators and denominators. HIV surveillance classifies males as MSM if the individual reports any sex with a man since 1977, whereas MSM denominators were defined as having male-male sex in the previous 5 years [1, 17]. This shorter time frame is more informative for assessing public-health-actionable HIV burden, compared with a lifetime definition of male-male sex. However, to the extent that some men living with HIV were classified as MSM at the time of diagnoses but may not have engaged in sexual activity in the previous 5 years, some inflation of prevalence may be observed. There is also a potential age mismatch between the HIV case surveillance-based numerator data and the MSM population size denominators. Publicly available HIV surveillance data reports MSM cases for all persons 13 years of age and older, whereas MSM population size methodologies are for MSM 18 years of age and older. This potentially inflates MSM prevalence. However, persons aged 13-19 years compose only 0.96% of all US HIV diagnoses and diagnosed HIV prevalence is low among MSM aged 13-17 years, so the extent of this bias is likely minimal [1, 32].

### Conclusions

Surveillance data have been described as the conscience of the HIV epidemic [33], and the new insights provided here on the rates of HIV prevalence and new diagnoses for US MSM constitute a call of conscience for heightened responses and improved monitoring of HIV epidemics among MSM, especially in the South. Across the United States, MSM are affected by HIV at rates that are orders of magnitude higher than for other Americans. This health disparity is even more pronounced in the South. There is a need for increased resources for HIV prevention, treatment, and care for MSM. In the South, this must include expansion of access to health care through Medicaid expansion under the Affordable Care Act; increased access to comprehensive HIV prevention services, including for preexposure prophylaxis (PrEP); and increased resources for programs to support immediate referrals for antiretroviral therapy for those who are newly diagnosed with HIV. Despite the disproportionate impact of HIV in the South, PrEP uptake among MSM is lower in the South than in other geographic areas [34, 35]. In terms of monitoring, we believe that our analysis illustrates the power of having denominators available to characterize health outcomes of sexual minority groups, and we join the Institute of Medicine’s call [36] to collect data on sexual orientation and gender identity in federal data collections and electronic health records, as well as to consider collecting such data in the United States Census. The health disparities in HIV for US MSM illustrated in this report are intolerable, and we call for urgent action to meet the treatment and care needs of those MSM living with HIV and to support all available evidence-based approaches to prevent new infections.

## Appendix

Multimedia Appendix 1 Supplement with additional data and sensitivity analyses.

## Abbreviations

ACS: American Community Survey
CDC: Centers for Disease Control and Prevention
HIV: human immunodeficiency virus
MSA: metropolitan statistical area
MSM: men who have sex with men
NHANES: National Health and Nutrition Examination Survey
NHAS: United States National HIV/AIDS Strategy
NHBS: National HIV Behavioral Surveillance System
PrEP: preexposure prophylaxis
