# Supplementary material for: Rates of Prevalent HIV Infection, Prevalent Diagnoses, and New Diagnoses Among Men Who Have Sex With Men in US States, Metropolitan Statistical Areas, and Counties, 2012-2013
Source: JMIR Public Health Surveill. 2016 May 17;2(1):e22. doi: 10.2196/publichealth.5684 (PMC4887662; doi:10.2196/publichealth.5684)
Supplement: Multimedia Appendix 1 [file publichealth_v2i1e22_app1.pdf]

Supplement

Rates of prevalent HIV infection, prevalent diagnoses and new diagnoses among men who have sex with men (MSM) in US states, metropolitan statistical areas, and counties, 2012-2013

Eli S. Rosenberg, PhD, Jeremy A. Grey, PhD, Travis H. Sanchez, DVM, Patrick S. Sullivan, PhD DVM

Part 1. Number of MSM, 2012

This manuscript used estimates of the county-level population sizes of men who have sex with men (MSM) in 2012 and 2013. The results for 2013 are included in the companion article on our method [1]. Here, we provide estimates for 2012 at the state and metropolitan statistical area (MSA) levels (also referred to as core-based statistical area [CBSA] level in the companion article).

Supplement Table 1. Estimated population size of men who have sex with men in 50 states and the District of Columbia, using housing and population estimates from the American Community Survey, 2008-2012.

	Adult males	MSM	
**State**	***N***	***n***	**% of males**
Alabama	1,739,878	40,812	2.3%
Alaska	273,669	5,162	1.9%
Arizona	2,357,342	107,515	4.6%
Arkansas	1,068,010	18,909	1.8%
California	13,810,868	785,954	5.7%
Colorado	1,904,108	70,287	3.7%
Connecticut	1,323,562	43,525	3.3%
Delaware	331,320	13,193	4.0%
District of Columbia	233,939	35,110	15.0%
Florida	7,186,086	331,070	4.6%
Georgia	3,478,235	130,214	3.7%
Hawaii	527,813	15,751	3.0%
Idaho	567,187	9,687	1.7%
Illinois	4,701,376	201,596	4.3%
Indiana	2,371,363	70,228	3.0%
Iowa	1,136,654	20,774	1.8%
Kansas	1,045,398	22,687	2.2%
Kentucky	1,609,713	45,741	2.8%
Louisiana	1,646,198	41,220	2.5%
Maine	509,723	15,025	2.9%

Maryland	2,109,864	82,629	3.9%
Massachusetts	2,450,324	112,995	4.6%
Michigan	3,658,367	112,434	3.1%
Minnesota	1,982,819	79,645	4.0%
Mississippi	1,055,795	19,151	1.8%
Missouri	2,204,131	72,415	3.3%
Montana	382,786	6,530	1.7%
Nebraska	672,249	12,677	1.9%
Nevada	1,025,612	51,429	5.0%
New Hampshire	503,623	13,997	2.8%
New Jersey	3,231,850	130,952	4.1%
New Mexico	752,990	16,980	2.3%
New York	7,184,245	373,806	5.2%
North Carolina	3,486,140	101,062	2.9%
North Dakota	264,770	3,969	1.5%
Ohio	4,241,771	139,189	3.3%
Oklahoma	1,380,741	37,671	2.7%
Oregon	1,456,607	60,528	4.2%
Pennsylvania	4,765,333	159,785	3.4%
Rhode Island	394,203	23,182	5.9%
South Carolina	1,703,151	33,896	2.0%
South Dakota	305,101	5,411	1.8%
Tennessee	2,332,882	74,852	3.2%
Texas	9,007,898	365,088	4.1%
Utah	944,257	32,522	3.4%
Vermont	242,270	6,942	2.9%
Virginia	2,989,024	111,842	3.7%
Washington	2,552,526	112,627	4.4%
West Virginia	712,946	12,556	1.8%
Wisconsin	2,139,759	58,258	2.7%
Wyoming	217,176	3,290	1.5%
**Total**	**114,173,652**	**4,452,772**	**3.9%**

Supplement Table 2. Estimated population size of men who have sex with men in 104 metropolitan statistical areas with HIV surveillance data, using housing and population estimates from the American Community Survey, 2008-2012.

	Adult males	MSM	
**Metropolitan statistical area**	***n***	***n***	**% of males**
Akron, OH	260,901	5,810	2.2%
Albany--Schenectady--Troy, NY	330,126	9,294	2.8%
Albuquerque, NM	325,150	8,608	2.6%
Allentown--Bethlehem--Easton, PA--NJ	304,919	6,637	2.2%
Atlanta--Sandy Springs--Roswell, GA	1,871,931	99,826	5.3%
Augusta--Richmond County, GA--SC	204,039	3,464	1.7%
Austin--Round Rock, TX	645,051	39,949	6.2%
Bakersfield, CA	303,162	5,923	2.0%
Baltimore--Columbia--Towson, MD	990,429	44,827	4.5%
Baton Rouge, LA	292,975	6,200	2.1%
Birmingham--Hoover, AL	405,899	16,451	4.1%
Boise City, ID	221,426	3,829	1.7%
Boston--Cambridge--Newton, MA--NH	1,707,425	93,254	5.5%
Bridgeport--Stamford--Norwalk, CT	330,908	8,315	2.5%
Buffalo--Cheektowaga--Niagara Falls, NY	423,522	21,926	5.2%
Cape Coral--Fort Myers, FL	243,975	7,157	2.9%
Charleston--North Charleston, SC	248,282	5,539	2.2%
Charlotte--Concord--Gastonia, NC--SC	793,084	34,822	4.4%
Chattanooga, TN--GA	195,386	4,364	2.2%
Chicago--Naperville--Elgin, IL--IN--WI	3,417,204	177,280	5.2%
Cincinnati, OH--KY--IN	764,794	30,977	4.1%
Cleveland--Elyria, OH	753,541	33,700	4.5%
Colorado Springs, CO	237,290	4,556	1.9%
Columbia, SC	282,061	6,384	2.3%
Columbus, OH	695,762	37,445	5.4%
Dallas--Fort Worth--Arlington, TX	2,272,239	131,843	5.8%
Dayton, OH	293,801	6,582	2.2%
Deltona--Daytona Beach--Ormond Beach, FL	230,773	5,612	2.4%
Denver--Aurora--Lakewood, CO	946,541	53,532	5.7%
Des Moines--West Des Moines, IA	204,551	4,820	2.4%
Detroit--Warren--Dearborn, MI	1,554,107	62,835	4.0%
Durham--Chapel Hill, NC	185,349	5,490	3.0%
El Paso, TX	268,100	4,353	1.6%
Fresno, CA	323,309	7,496	2.3%
Grand Rapids--Wyoming, MI	357,276	16,825	4.7%
Greensboro--High Point, NC	261,145	5,800	2.2%
Greenville--Anderson--Mauldin, SC	303,073	5,620	1.9%

Harrisburg--Carlisle, PA	206,297	5,698	2.8%
Hartford--West Hartford--East Hartford, CT	452,424	22,723	5.0%
Honolulu (Urban), HI	371,128	10,392	2.8%
Houston--The Woodlands--Sugar Land, TX	2,107,885	102,173	4.8%
Indianapolis--Carmel--Anderson, IN	673,477	32,882	4.9%
Jackson, MS	197,416	4,067	2.1%
Jacksonville, FL	493,776	22,767	4.6%
Kansas City, MO--KS	721,757	33,127	4.6%
Knoxville, TN	316,231	6,127	1.9%
Lakeland--Winter Haven, FL	223,966	5,511	2.5%
Lancaster, PA	188,228	4,637	2.5%
Las Vegas--Henderson--Paradise, NV	734,840	45,828	6.2%
Little Rock--North Little Rock--Conway, AR	253,254	5,178	2.0%
Los Angeles--Long Beach--Anaheim, CA	4,741,785	308,066	6.5%
Louisville--Jefferson County, KY--IN	452,825	22,743	5.0%
Madison, WI	233,189	6,417	2.8%
McAllen--Edinburg--Mission, TX	241,482	4,286	1.8%
Memphis, TN--MS--AR	457,558	21,028	4.6%
Miami--Fort Lauderdale--West Palm Beach, FL	2,096,754	133,591	6.4%
Milwaukee--Waukesha--West Allis, WI	561,587	25,933	4.6%
Minneapolis--St. Paul--Bloomington, MN--WI	1,232,429	67,872	5.5%
Modesto, CA	179,312	3,217	1.8%
Nashville--Davidson--Murfreesboro--Franklin, TN	610,381	30,347	5.0%
New Haven--Milford, CT	316,784	7,389	2.3%
New Orleans--Metairie, LA	435,817	20,395	4.7%
New York--Newark--Jersey City, NY--NJ--PA	7,165,159	395,928	5.5%
North Port--Sarasota--Bradenton, FL	274,444	8,108	3.0%
Ogden--Clearfield, UT	199,809	3,625	1.8%
Oklahoma City, OK	458,782	21,265	4.6%
Omaha--Council Bluffs, NE--IA	311,192	7,574	2.4%
Orlando--Kissimmee--Sanford, FL	795,885	45,547	5.7%
Oxnard--Thousand Oaks--Ventura, CA	301,053	7,522	2.5%
Palm Bay--Melbourne--Titusville, FL	211,429	5,032	2.4%
Philadelphia--Camden--Wilmington, PA--NJ--DE--MD	2,171,443	100,373	4.6%
Phoenix--Mesa--Scottsdale, AZ	1,530,468	89,126	5.8%
Pittsburgh, PA	896,941	37,891	4.2%
Portland--South Portland, ME	194,947	7,562	3.9%
Portland--Vancouver--Hillsboro, OR--WA	833,286	52,287	6.3%
Providence--Warwick, RI--MA	597,633	31,001	5.2%
Provo--Orem, UT	169,367	2,368	1.4%
Raleigh, NC	403,724	20,135	5.0%
Richmond, VA	441,102	17,772	4.0%
Riverside--San Bernardino--Ontario, CA	1,487,933	93,321	6.3%

Rochester, NY	400,877	20,825	5.2%
Sacramento--Roseville--Arden-Arcade, CA	783,062	44,804	5.7%
Salt Lake City, UT	384,280	22,669	5.9%
San Antonio--New Braunfels, TX	763,142	35,488	4.7%
San Diego--Carlsbad--San Marcos, CA	1,185,465	79,742	6.7%
San Francisco--Oakland--Hayward, CA	1,675,079	149,806	8.9%
San Jose--Sunnyvale--Santa Clara, CA	697,953	37,748	5.4%
Scranton--Wilkes-Barre--Hazelton, PA	215,374	3,739	1.7%
Seattle--Tacoma--Bellevue, WA	1,319,369	81,998	6.2%
Spokane--Spokane Valley, WA	198,082	5,264	2.7%
Springfield, MA	228,149	5,176	2.3%
St. Louis, MO--IL	1,008,456	39,036	3.9%
Stockton--Lodi, CA	239,028	5,130	2.1%
Syracuse, NY	244,499	6,150	2.5%
Tampa--St. Petersburg--Clearwater, FL	1,051,763	62,239	5.9%
Toledo, OH	222,959	4,570	2.0%
Tucson, AZ	367,451	10,401	2.8%
Tulsa, OK	337,751	7,307	2.2%
Virginia Beach--Norfolk--Newport News, VA--NC	622,546	24,493	3.9%
Washington--Arlington--Alexandria, DC--VA--MD--WV	2,068,780	118,961	5.8%
Wichita, KS	225,215	4,019	1.8%
Winston-Salem, NC	230,211	4,913	2.1%
Worcester, MA--CT	342,863	8,709	2.5%
Youngstown--Warren--Boardman, OH--PA	211,666	3,549	1.7%

Part 2. Sensitivity Analyses

Methods

The denominators for our analyses were derived using a method of estimating the county-level population sizes of men who have sex with men (MSM) [1]. In order to examine the influence of this estimation method on our findings, we conducted analyses using two additional sets of denominators from methods based on previous studies [2, 3]. For the following tables, we have labeled these methods Method 1, Method 2, and Method 3, in order of increasing complexity. The first, Method 1, multiplies a standard percentage of adult men who have had sex with another man in the past five years by the number of adult men in each county to determine the number of MSM. For this value, we used 3.9%, as reported in Purcell et al. The second method (Method 2) uses the stratified percentages of adult men who have had sex with another man in the past year at four levels of urbanicity: *large central metropolitan counties* (4.4%); *large fringe metropolitan counties* (2.5%); *small or medium metropolitan counties* (1.4%); and *non-metropolitan counties* (1.1%). These percentages are multiplied by the number of adult males in counties of the corresponding urbanicity levels to obtain the number of men who had sex with another man in the past year. These population sizes are then scaled up to achieve the national 5-year male-male sex estimate from Method 1, or 3.9% of the US adult male population. Finally, Method 3 applies the method reported in Grey et al. [1], which uses the assumed national percentage of US men who have had sex with another man in the past five years [3], the urbanicity-specific percentage of US

men who have had sex with another man in the past year [2], and the relative representation of same-sex male households in an area, as used by Lieb et al [4]. A summary of the major components of the three models is provided in Supplement Table 3.

Supplement Table 3. Major components of three methods to estimate the county-level population sizes of men who have sex with men in the US, using data from the American Community Survey

	Method		
**Component**	**1**	**2**	**3**
3.9% of adult men in the US have had sex with another man in the past five years	X	X	X
Variation of the percentage of adult men who have had sex with another man by urbanicity strata		X	X
Within urbanicity strata, the percentage of adult men who have sex with men varies according to the relative representation of same-sex male households			X

Results

Each method was used to generate the outcomes reported in Tables 2 through 4 of the manuscript. The following tables present findings according to the three methods, with each rate as its own table. Thus, Table 2 is presented as Supplement Tables 4a (state-level prevalences of HIV diagnosis among MSM), 4b (state-level rates of new HIV diagnosis among all MSM), and 4c (state-level rates of new HIV diagnosis among MSM without an HIV diagnosis); Table 3 is presented as Supplement Tables 5a (state-level prevalence of HIV among MSM) and 5b (state-level prevalence of undiagnosed HIV among MSM); and Table 4 is presented as Supplement Tables 6a (CBSA-level prevalence of HIV diagnosis among MSM), 6b (CBSA-level rates of new diagnosis among all MSM), and 6c (CBSA-level rates of new diagnosis among MSM without an HIV diagnosis).

Supplement Table 4a. Prevalence of HIV diagnoses among men who have sex with men, US states and District of Columbia, 2012

		Rate per 100 MSM		
**State**	**n**	**Method 1**	**Method 2**	**Method 3**
Alabama	6,442	9.49	13.05	15.78
Alaska	335	3.14	6.10	6.49
Arizona	8,748	9.52	7.47	8.14
Arkansas	2,843	6.83	13.30	15.04
California	85,307	15.84	11.60	10.85
Colorado	8,028	10.81	12.76	11.42
Connecticut	3,178	6.16	6.97	7.30
Delaware	1,115	8.63	10.59	8.45
District of Columbia	7,360	80.67	46.29	20.96
Florida	47,520	16.96	15.70	14.35
Georgia	24,101	17.77	21.15	18.51
Hawaii	1,758	8.54	16.03	11.16
Idaho	477	2.16	4.21	4.92
Illinois	20,495	11.18	9.69	10.17

Indiana	5,876	6.35	7.73	8.37
Iowa	1,133	2.56	5.08	5.45
Kansas	1,723	4.23	6.63	7.59
Kentucky	3,697	5.89	7.68	8.08
Louisiana	8,954	13.95	19.71	21.72
Maine	771	3.88	7.69	5.13
Maryland	11,052	13.43	13.24	13.38
Massachusetts	8,181	8.56	9.00	7.24
Michigan	9,377	6.57	7.03	8.34
Minnesota	4,416	5.71	5.58	5.54
Mississippi	4,469	10.85	20.70	23.34
Missouri	7,994	9.30	10.59	11.04
Montana	239	1.60	3.35	3.66
Nebraska	1,015	3.87	7.59	8.01
Nevada	5,070	12.68	9.09	9.86
New Hampshire	621	3.16	4.89	4.44
New Jersey	13,402	10.63	9.61	10.23
New Mexico	1,729	5.89	11.44	10.18
New York	54,606	19.49	15.44	14.61
North Carolina	13,202	9.71	12.09	13.06
North Dakota	132	1.28	2.59	3.33
Ohio	12,259	7.41	7.83	8.81
Oklahoma	3,293	6.12	7.67	8.74
Oregon	3,673	6.47	7.31	6.07
Pennsylvania	12,477	6.71	7.33	7.81
Rhode Island	949	6.17	4.30	4.09
South Carolina	7,332	11.04	19.51	21.63
South Dakota	196	1.65	3.36	3.62
Tennessee	9,198	10.11	11.42	12.29
Texas	42,973	12.23	10.45	11.77
Utah	1,532	4.16	4.12	4.71
Vermont	275	2.91	6.12	3.96
Virginia	11,888	10.20	10.66	10.63
Washington	7,681	7.72	7.59	6.82
West Virginia	930	3.34	6.43	7.41
Wisconsin	3,388	4.06	5.23	5.82
Wyoming	133	1.57	3.34	4.04
**50 US States & Washington, DC**	**493,453**	**11.08**	**11.08**	**11.08**

Supplement Table 4b. Rates of new diagnoses among men who have sex with men, US states and District of Columbia, 2013

		Rate per 100 MSM		
**State**	**n**	**Method 1**	**Method 2**	**Method 3**
Alabama	442	0.65	0.89	1.09
Alaska	12	0.11	0.22	0.24
Arizona	513	0.55	0.43	0.46
Arkansas	192	0.46	0.89	1.00
California	3,860	0.71	0.52	0.49
Colorado	241	0.32	0.38	0.33
Connecticut	188	0.36	0.41	0.43
Delaware	60	0.46	0.56	0.46
District of Columbia	313	3.35	1.92	0.85
Florida	2,711	0.95	0.88	0.80
Georgia	1,708	1.24	1.48	1.30
Hawaii	78	0.37	0.70	0.51
Idaho	15	0.07	0.13	0.15
Illinois	1,273	0.69	0.60	0.64
Indiana	332	0.36	0.43	0.47
Iowa	77	0.17	0.34	0.37
Kansas	109	0.27	0.42	0.48
Kentucky	260	0.41	0.54	0.55
Louisiana	730	1.12	1.58	1.76
Maine	21	0.11	0.21	0.14
Maryland	762	0.91	0.90	0.90
Massachusetts	443	0.46	0.48	0.40
Michigan	547	0.38	0.41	0.48
Minnesota	202	0.26	0.25	0.24
Mississippi	316	0.76	1.45	1.66
Missouri	341	0.39	0.45	0.48
Montana	19	0.13	0.26	0.30
Nebraska	51	0.19	0.38	0.39
Nevada	329	0.81	0.58	0.64
New Hampshire	21	0.11	0.16	0.15
New Jersey	790	0.62	0.56	0.60
New Mexico	102	0.34	0.67	0.57
New York	2,264	0.80	0.63	0.61
North Carolina	859	0.62	0.77	0.83
North Dakota	13	0.12	0.25	0.29
Ohio	767	0.46	0.49	0.53
Oklahoma	236	0.43	0.54	0.63
Oregon	159	0.28	0.31	0.26
Pennsylvania	739	0.39	0.43	0.45
Rhode Island	54	0.35	0.24	0.23
South Carolina	452	0.67	1.19	1.24
South Dakota	9	0.07	0.15	0.17
Tennessee	563	0.61	0.69	0.76

Texas	3,129	0.87	0.75	0.84
Utah	72	0.19	0.19	0.22
Vermont	12	0.13	0.27	0.17
Virginia	683	0.58	0.60	0.61
Washington	325	0.32	0.32	0.29
West Virginia	46	0.16	0.32	0.35
Wisconsin	190	0.23	0.29	0.32
Wyoming	11	0.13	0.27	0.34
**50 US States & Washington, DC**	**27,641**	**0.61**	**0.61**	**0.61**

Supplement Table 4c. Rates of new diagnoses among men who have sex with men and who do not have an HIV diagnosis, US states and District of Columbia, 2013

		Rate per 100 MSM without an HIV diagnosis		
**State**	**n**	**Method 1**	**Method 2**	**Method 3**
Alabama	442	0.71	1.02	1.29
Alaska	12	0.11	0.23	0.25
Arizona	513	0.61	0.47	0.50
Arkansas	192	0.49	1.03	1.17
California	3,860	0.84	0.59	0.55
Colorado	241	0.36	0.43	0.37
Connecticut	188	0.38	0.44	0.47
Delaware	60	0.50	0.63	0.50
District of Columbia	313	15.68	3.51	1.06
Florida	2,711	1.15	1.05	0.93
Georgia	1,708	1.51	1.87	1.59
Hawaii	78	0.41	0.83	0.57
Idaho	15	0.07	0.14	0.16
Illinois	1,273	0.78	0.66	0.71
Indiana	332	0.38	0.47	0.52
Iowa	77	0.18	0.36	0.39
Kansas	109	0.28	0.45	0.51
Kentucky	260	0.44	0.58	0.60
Louisiana	730	1.30	1.96	2.24
Maine	21	0.11	0.23	0.15
Maryland	762	1.05	1.04	1.04
Massachusetts	443	0.50	0.53	0.43
Michigan	547	0.41	0.44	0.52
Minnesota	202	0.27	0.27	0.26
Mississippi	316	0.85	1.83	2.18
Missouri	341	0.43	0.50	0.54
Montana	19	0.13	0.27	0.31
Nebraska	51	0.20	0.41	0.42
Nevada	329	0.93	0.64	0.71

New Hampshire	21	0.11	0.17	0.16
New Jersey	790	0.70	0.62	0.66
New Mexico	102	0.36	0.75	0.63
New York	2,264	0.99	0.75	0.72
North Carolina	859	0.69	0.88	0.96
North Dakota	13	0.12	0.26	0.30
Ohio	767	0.50	0.53	0.58
Oklahoma	236	0.46	0.59	0.69
Oregon	159	0.30	0.34	0.27
Pennsylvania	739	0.42	0.47	0.49
Rhode Island	54	0.37	0.26	0.24
South Carolina	452	0.75	1.47	1.56
South Dakota	9	0.08	0.16	0.18
Tennessee	563	0.68	0.78	0.87
Texas	3,129	0.99	0.83	0.95
Utah	72	0.20	0.20	0.23
Vermont	12	0.13	0.28	0.18
Virginia	683	0.64	0.67	0.68
Washington	325	0.35	0.34	0.31
West Virginia	46	0.17	0.34	0.38
Wisconsin	190	0.24	0.31	0.34
Wyoming	11	0.13	0.28	0.36
**50 US States & Washington, DC**	**27,641**	**0.69**	**0.69**	**0.69**

Supplement Table 5a. Prevalence of HIV infection among men who have sex with men, by US state, 2012

		Rate per 100 MSM		
**State**	**n**	**Method 1**	**Method 2**	**Method 3**
Alabama	7,900	11.64	16.00	19.36
Alaska *	410	3.84	7.47	7.94
Arizona	10,500	11.42	8.97	9.77
Arkansas	3,500	8.40	16.37	18.51
California	134,400	24.95	18.27	17.10
Colorado	8,900	11.98	14.15	12.66
Connecticut	4,600	8.91	10.08	10.57
Delaware	1,600	12.38	15.20	12.13
District of Columbia	11,300	123.85	71.06	32.18
Florida	60,500	21.59	19.99	18.27
Georgia	33,100	24.40	29.04	25.42
Hawaii	2,500	12.14	22.80	15.87
Idaho *	630	2.85	5.56	6.50
Illinois	27,800	15.16	13.14	13.79

Indiana	6,900	7.46	9.08	9.83
Iowa	1,600	3.61	7.17	7.70
Kansas	2,200	5.40	8.47	9.70
Kentucky	5,300	8.44	11.02	11.59
Louisiana	10,700	16.67	23.56	25.96
Maine *	1,200	6.04	11.96	7.99
Maryland	16,200	19.69	19.41	19.61
Massachusetts	12,200	12.77	13.42	10.80
Michigan	10,900	7.64	8.17	9.69
Minnesota	5,200	6.72	6.57	6.53
Mississippi	5,400	13.11	25.02	28.20
Missouri	9,100	10.59	12.05	12.57
Montana *	420	2.81	5.90	6.43
Nebraska *	1,300	4.96	9.72	10.25
Nevada	6,500	16.25	11.66	12.64
New Hampshire *	950	4.84	7.48	6.79
New Jersey	16,800	13.33	12.04	12.83
New Mexico	2,400	8.17	15.88	14.13
New York	75,900	27.09	21.46	20.30
North Carolina	16,100	11.84	14.74	15.93
North Dakota *	190	1.84	3.72	4.79
Ohio	14,800	8.95	9.45	10.63
Oklahoma	4,100	7.61	9.54	10.88
Oregon	5,800	10.21	11.54	9.58
Pennsylvania	16,100	8.66	9.46	10.08
Rhode Island	1,100	7.15	4.99	4.75
South Carolina	9,500	14.30	25.28	28.03
South Dakota	200	1.68	3.43	3.70
Tennessee	11,000	12.09	13.66	14.70
Texas	62,400	17.76	15.17	17.09
Utah	1,700	4.62	4.57	5.23
Vermont *	520	5.50	11.57	7.49
Virginia	13,500	11.58	12.11	12.07
Washington	10,400	10.45	10.28	9.23
West Virginia *	1,200	4.32	8.29	9.56
Wisconsin	4,000	4.79	6.17	6.87
Wyoming *	180	2.13	4.52	5.47
**50 US States & Washington, DC §**	**666,900**	**15.08**	**15.08**	**15.08**

* Counts indicated as numerically unstable, per the source US Centers for Disease Control and Prevention report [5]

§ Total counts calculated by different methodology than used for jurisdictions and thus do not sum to column totals, per the source US Centers for Disease Control and Prevention report [5]

Supplement Table 5b. Prevalence of undiagnosed HIV infection among men who have sex with men, by US state, 2012

		Rate per 100 MSM		
**State**	**n**	**Method 1**	**Method 2**	**Method 3**
Alabama	1,600	2.36	3.24	3.92
Alaska *	20	0.19	0.36	0.39
Arizona	1,200	1.31	1.02	1.12
Arkansas	800	1.92	3.74	4.23
California	16,400	3.04	2.23	2.09
Colorado	950	1.28	1.51	1.35
Connecticut	710	1.38	1.56	1.63
Delaware	240	1.86	2.28	1.82
District of Columbia	1,400	15.34	8.80	3.99
Florida	8,100	2.89	2.68	2.45
Georgia	6,900	5.09	6.05	5.30
Hawaii	220	1.07	2.01	1.40
Idaho *	80	0.36	0.71	0.83
Illinois	5,300	2.89	2.51	2.63
Indiana	1,000	1.08	1.32	1.42
Iowa	330	0.74	1.48	1.59
Kansas	380	0.93	1.46	1.67
Kentucky	890	1.42	1.85	1.95
Louisiana	2,700	4.21	5.94	6.55
Maine *	90	0.45	0.90	0.60
Maryland	3,900	4.74	4.67	4.72
Massachusetts	2,000	2.09	2.20	1.77
Michigan	1,900	1.33	1.42	1.69
Minnesota	770	1.00	0.97	0.97
Mississippi	1,200	2.91	5.56	6.27
Missouri	1,500	1.74	1.99	2.07
Montana *	30	0.20	0.42	0.46
Nebraska *	190	0.72	1.42	1.50
Nevada	1,000	2.50	1.79	1.94
New Hampshire *	120	0.61	0.95	0.86
New Jersey	3,700	2.94	2.65	2.83
New Mexico	280	0.95	1.85	1.65
New York	7,700	2.75	2.18	2.06
North Carolina	2,600	1.91	2.38	2.57
North Dakota *	20	0.19	0.39	0.50
Ohio	3,100	1.87	1.98	2.23
Oklahoma	740	1.37	1.72	1.96
Oregon	850	1.50	1.69	1.40

Pennsylvania	2,700	1.45	1.59	1.69
Rhode Island	200	1.30	0.91	0.86
South Carolina	2,000	3.01	5.32	5.90
South Dakota	30	0.25	0.51	0.55
Tennessee	1,800	1.98	2.24	2.40
Texas	12,100	3.44	2.94	3.31
Utah	250	0.68	0.67	0.77
Vermont *	0	0.00	0.00	0.00
Virginia	2,000	1.72	1.79	1.79
Washington	1,300	1.31	1.28	1.15
West Virginia *	200	0.72	1.38	1.59
Wisconsin	650	0.78	1.00	1.12
Wyoming *	40	0.47	1.00	1.22
**50 US States & Washington, DC §**	**98,700**	**2.34**	**2.34**	**2.34**

* Counts indicated as numerically unstable, per the source US Centers for Disease Control and Prevention report [5]

§ Total counts calculated by different methodology than used for jurisdictions and thus do not sum to column totals, per the source US Centers for Disease Control and Prevention report [5]

Supplement Table 6a. Prevalence of HIV diagnoses among men who have sex with men, 104 US metropolitan statistical areas, 2012

		Rate per 100 MSM		
**Metropolitan Statistical Area**	**n**	**Method 1**	**Method 2**	**Method 3**
Akron, OH	529	5.20	9.38	9.10
Albany--Schenectady--Troy, NY	778	6.04	10.90	8.37
Albuquerque, NM	878	6.92	12.49	10.20
Allentown--Bethlehem--Easton, PA--NJ	405	3.41	6.14	6.10
Atlanta--Sandy Springs--Roswell, GA	16,404	22.47	19.93	16.43
Augusta--Richmond County, GA--SC	920	11.56	20.85	26.56
Austin--Round Rock, TX	3,201	12.72	8.76	8.01
Bakersfield, CA	692	5.85	10.55	11.68
Baltimore--Columbia--Towson, MD	5,631	14.58	12.56	12.56
Baton Rouge, LA	1,575	13.78	24.86	25.40
Birmingham--Hoover, AL	2,196	13.87	9.76	13.35
Boise City, ID	271	3.14	5.66	7.08
Boston--Cambridge--Newton, MA--NH	6,216	9.33	8.37	6.67
Bridgeport--Stamford--Norwalk, CT	841	6.52	11.75	10.11
Buffalo--Cheektowaga--Niagara Falls, NY	937	5.67	3.55	4.27
Cape Coral--Fort Myers, FL	618	6.49	11.71	8.63
Charleston--North Charleston, SC	1,112	11.48	20.71	20.08
Charlotte--Concord--Gastonia, NC--SC	3,567	11.53	8.85	10.24

Chattanooga, TN--GA	657	8.62	15.55	15.06
Chicago--Naperville--Elgin, IL--IN--WI	18,183	13.64	9.70	10.26
Cincinnati, OH--KY--IN	2,198	7.37	5.78	7.10
Cleveland--Elyria, OH	2,824	9.61	6.63	8.38
Colorado Springs, CO	522	5.64	10.17	11.46
Columbia, SC	1,893	17.21	31.03	29.65
Columbus, OH	3,326	12.26	8.45	8.88
Dallas--Fort Worth--Arlington, TX	14,654	16.54	10.54	11.11
Dayton, OH	911	7.95	14.34	13.84
Deltona--Daytona Beach--Ormond Beach, FL	709	7.88	14.21	12.63
Denver--Aurora--Lakewood, CO	6,428	17.41	14.78	12.01
Des Moines--West Des Moines, IA	361	4.53	8.16	7.49
Detroit--Warren--Dearborn, MI	5,834	9.63	7.40	9.28
Durham--Chapel Hill, NC	959	13.27	23.92	17.47
El Paso, TX	1,242	11.88	21.42	28.53
Fresno, CA	1,034	8.20	14.79	13.79
Grand Rapids--Wyoming, MI	587	4.21	2.92	3.49
Greensboro--High Point, NC	1,217	11.95	21.55	20.98
Greenville--Anderson--Mauldin, SC	903	7.64	13.78	16.07
Harrisburg--Carlisle, PA	534	6.64	11.97	9.37
Hartford--West Hartford--East Hartford, CT	1,032	5.85	3.81	4.54
Houston--The Woodlands--Sugar Land, TX	12,861	15.64	10.36	12.59
Indianapolis--Carmel--Anderson, IN	2,951	11.24	8.33	8.97
Jackson, MS	1,606	20.86	37.62	39.49
Jacksonville, FL	2,437	12.65	8.59	10.70
Kansas City, MO--KS	3,309	11.76	9.47	9.99
Knoxville, TN	757	6.14	11.07	12.36
Lakeland--Winter Haven, FL	591	6.77	12.20	10.72
Lancaster, PA	270	3.68	6.63	5.82
Las Vegas--Henderson--Paradise, NV	4,121	14.38	8.25	8.99
Little Rock--North Little Rock--Conway, AR	1,208	12.23	22.06	23.33
Los Angeles--Long Beach--Anaheim, CA	39,798	21.52	12.35	12.92
Louisville--Jefferson County, KY--IN	1,874	10.61	7.38	8.24
Madison, WI	427	4.70	8.47	6.65
McAllen--Edinburg--Mission, TX	605	6.42	11.58	14.12
Memphis, TN--MS--AR	3,704	20.76	13.70	17.61
Miami--Fort Lauderdale--West Palm Beach, FL	23,407	28.62	21.57	17.52
Milwaukee--Waukesha--West Allis, WI	1,836	8.38	5.81	7.08
Minneapolis--St. Paul--Bloomington, MN--WI	3,890	8.09	5.91	5.73
Modesto, CA	312	4.46	8.05	9.70
Nashville--Davidson--Murfreesboro--Franklin, TN	2,974	12.49	9.76	9.80
New Haven--Milford, CT	870	7.04	12.70	11.77
New Orleans--Metairie, LA	4,020	23.65	19.54	19.71

New York--Newark--Jersey City, NY--NJ--PA	57,861	20.71	15.01	14.61
North Port--Sarasota--Bradenton, FL	795	7.43	13.39	9.80
Ogden--Clearfield, UT	185	2.37	4.28	5.10
Oklahoma City, OK	1,599	8.94	6.31	7.52
Omaha--Council Bluffs, NE--IA	704	5.80	10.46	9.30
Orlando--Kissimmee--Sanford, FL	5,555	17.90	12.82	12.20
Oxnard--Thousand Oaks--Ventura, CA	610	5.20	9.37	8.11
Palm Bay--Melbourne--Titusville, FL	539	6.54	11.79	10.71
Philadelphia--Camden--Wilmington, PA--NJ--DE--MD	9,127	10.78	9.14	9.09
Phoenix--Mesa--Scottsdale, AZ	6,511	10.91	6.53	7.31
Pittsburgh, PA	1,785	5.10	3.70	4.71
Portland--South Portland, ME	416	5.47	9.87	5.50
Portland--Vancouver--Hillsboro, OR--WA	3,043	9.36	7.49	5.82
Providence--Warwick, RI--MA	1,206	5.17	4.03	3.89
Provo--Orem, UT	87	1.32	2.38	3.67
Raleigh, NC	1,816	11.53	7.25	9.02
Richmond, VA	2,259	13.13	11.69	12.71
Riverside--San Bernardino--Ontario, CA	5,406	9.32	6.74	5.79
Rochester, NY	1,139	7.29	4.84	5.47
Sacramento--Roseville--Arden-Arcade, CA	2,191	7.17	4.84	4.89
St. Louis, MO--IL	4,526	11.51	10.67	11.59
Salt Lake City, UT	1,128	7.53	4.41	4.98
San Antonio--New Braunfels, TX	3,598	12.09	7.61	10.14
San Diego--Carlsbad--San Marcos, CA	9,170	19.83	11.38	11.50
San Francisco--Oakland--Hayward, CA	17,152	26.26	18.69	11.45
San Jose--Sunnyvale--Santa Clara, CA	2,248	8.26	4.80	5.96
Scranton--Wilkes-Barre--Hazelton, PA	212	2.52	4.55	5.67
Seattle--Tacoma--Bellevue, WA	5,938	11.54	8.12	7.24
Spokane--Spokane Valley, WA	254	3.29	5.93	4.82
Springfield, MA	556	6.25	11.27	10.74
Stockton--Lodi, CA	524	5.62	10.14	10.21
Syracuse, NY	487	5.11	9.21	7.92
Tampa--St. Petersburg--Clearwater, FL	5,504	13.42	8.55	8.84
Toledo, OH	516	5.93	10.70	11.29
Tucson, AZ	1,292	9.02	16.26	12.42
Tulsa, OK	1,029	7.81	14.09	14.08
Honolulu (Urban), HI	1,170	8.08	14.58	11.26
Virginia Beach--Norfolk--Newport News, VA--NC	3,527	14.53	11.12	14.40
Washington--Arlington--Alexandria, DC--VA--MD--WV	15,976	19.80	17.56	13.43
Wichita, KS	477	5.43	9.79	11.87
Winston-Salem, NC	790	8.80	15.87	16.08
Worcester, MA--CT	541	4.05	7.30	6.21
Youngstown--Warren--Boardman, OH--PA	322	3.90	7.03	9.07

Supplement Table 6b. Rates of new diagnoses among men who have sex with men, 104 US metropolitan statistical areas, 2013

		Rate per 100 MSM		
**Metropolitan Statistical Area**	**n**	**Method 1**	**Method 2**	**Method 3**
Akron, OH	36	0.35	0.63	0.58
Albany--Schenectady--Troy, NY	33	0.25	0.46	0.35
Albuquerque, NM	47	0.37	0.66	0.50
Allentown--Bethlehem--Easton, PA--NJ	30	0.25	0.45	0.46
Atlanta--Sandy Springs--Roswell, GA	1,393	1.88	1.67	1.36
Augusta--Richmond County, GA--SC	89	1.10	1.99	2.79
Austin--Round Rock, TX	256	0.99	0.68	0.63
Bakersfield, CA	72	0.60	1.09	1.13
Baltimore--Columbia--Towson, MD	515	1.32	1.14	1.14
Baton Rouge, LA	155	1.34	2.42	2.91
Birmingham--Hoover, AL	130	0.82	0.58	0.79
Boise City, ID	13	0.15	0.27	0.33
Boston--Cambridge--Newton, MA--NH	423	0.63	0.56	0.46
Bridgeport--Stamford--Norwalk, CT	50	0.38	0.69	0.61
Buffalo--Cheektowaga--Niagara Falls, NY	80	0.48	0.30	0.37
Cape Coral--Fort Myers, FL	72	0.74	1.34	0.93
Charleston--North Charleston, SC	84	0.85	1.53	1.46
Charlotte--Concord--Gastonia, NC--SC	285	0.90	0.69	0.80
Chattanooga, TN--GA	35	0.45	0.82	0.85
Chicago--Naperville--Elgin, IL--IN--WI	1,361	1.01	0.72	0.78
Cincinnati, OH--KY--IN	168	0.56	0.44	0.53
Cleveland--Elyria, OH	218	0.74	0.51	0.63
Colorado Springs, CO	18	0.19	0.34	0.36
Columbia, SC	99	0.89	1.60	1.43
Columbus, OH	229	0.83	0.58	0.56
Dallas--Fort Worth--Arlington, TX	1,019	1.13	0.72	0.76
Dayton, OH	61	0.53	0.96	0.89
Deltona--Daytona Beach--Ormond Beach, FL	55	0.61	1.10	0.95
Denver--Aurora--Lakewood, CO	198	0.52	0.45	0.36
Des Moines--West Des Moines, IA	28	0.34	0.62	0.57
Detroit--Warren--Dearborn, MI	390	0.64	0.49	0.61
Durham--Chapel Hill, NC	70	0.94	1.70	1.13
El Paso, TX	96	0.90	1.62	2.22
Fresno, CA	88	0.69	1.24	1.16
Grand Rapids--Wyoming, MI	31	0.22	0.15	0.19
Greensboro--High Point, NC	75	0.73	1.32	1.26
Greenville--Anderson--Mauldin, SC	80	0.67	1.21	1.42
Harrisburg--Carlisle, PA	30	0.37	0.67	0.52

Hartford--West Hartford--East Hartford, CT	73	0.41	0.27	0.33
Houston--The Woodlands--Sugar Land, TX	1,013	1.20	0.80	0.98
Indianapolis--Carmel--Anderson, IN	175	0.66	0.49	0.53
Jackson, MS	106	1.36	2.46	2.51
Jacksonville, FL	210	1.08	0.73	0.95
Kansas City, MO--KS	154	0.54	0.44	0.47
Knoxville, TN	49	0.39	0.71	0.81
Lakeland--Winter Haven, FL	49	0.56	1.00	0.88
Lancaster, PA	27	0.36	0.66	0.55
Las Vegas--Henderson--Paradise, NV	292	1.01	0.58	0.63
Little Rock--North Little Rock--Conway, AR	124	1.24	2.24	2.24
Los Angeles--Long Beach--Anaheim, CA	1,938	1.04	0.59	0.62
Louisville--Jefferson County, KY--IN	136	0.76	0.53	0.58
Madison, WI	27	0.29	0.53	0.41
McAllen--Edinburg--Mission, TX	66	0.68	1.23	1.60
Memphis, TN--MS--AR	257	1.43	0.94	1.18
Miami--Fort Lauderdale--West Palm Beach, FL	1,592	1.92	1.44	1.13
Milwaukee--Waukesha--West Allis, WI	98	0.44	0.31	0.37
Minneapolis--St. Paul--Bloomington, MN--WI	177	0.36	0.27	0.25
Modesto, CA	15	0.21	0.38	0.53
Nashville--Davidson--Murfreesboro--Franklin, TN	181	0.75	0.58	0.62
New Haven--Milford, CT	56	0.45	0.81	0.73
New Orleans--Metairie, LA	356	2.05	1.69	1.68
New York--Newark--Jersey City, NY--NJ--PA	3,007	1.07	0.77	0.76
North Port--Sarasota--Bradenton, FL	54	0.50	0.90	0.69
Ogden--Clearfield, UT	6	0.08	0.14	0.17
Oklahoma City, OK	155	0.85	0.60	0.74
Omaha--Council Bluffs, NE--IA	35	0.28	0.51	0.44
Orlando--Kissimmee--Sanford, FL	368	1.16	0.83	0.80
Oxnard--Thousand Oaks--Ventura, CA	53	0.45	0.81	0.73
Palm Bay--Melbourne--Titusville, FL	45	0.54	0.98	0.88
Philadelphia--Camden--Wilmington, PA--NJ--DE--MD	614	0.72	0.61	0.61
Phoenix--Mesa--Scottsdale, AZ	406	0.67	0.40	0.44
Pittsburgh, PA	109	0.31	0.22	0.28
Portland--South Portland, ME	12	0.16	0.28	0.17
Portland--Vancouver--Hillsboro, OR--WA	139	0.42	0.34	0.26
Providence--Warwick, RI--MA	94	0.40	0.31	0.30
Provo--Orem, UT	3	0.04	0.08	0.13
Raleigh, NC	141	0.87	0.55	0.67
Richmond, VA	123	0.70	0.63	0.70
Riverside--San Bernardino--Ontario, CA	370	0.63	0.45	0.39
Rochester, NY	67	0.43	0.28	0.34
Sacramento--Roseville--Arden-Arcade, CA	149	0.48	0.33	0.34

St. Louis, MO--IL	233	0.59	0.55	0.61
Salt Lake City, UT	45	0.29	0.17	0.19
San Antonio--New Braunfels, TX	365	1.20	0.75	0.96
San Diego--Carlsbad--San Marcos, CA	425	0.90	0.52	0.52
San Francisco--Oakland--Hayward, CA	643	0.97	0.69	0.44
San Jose--Sunnyvale--Santa Clara, CA	109	0.39	0.23	0.29
Scranton--Wilkes-Barre--Hazelton, PA	11	0.13	0.24	0.28
Seattle--Tacoma--Bellevue, WA	245	0.47	0.33	0.30
Spokane--Spokane Valley, WA	11	0.14	0.25	0.22
Springfield, MA	41	0.46	0.83	0.77
Stockton--Lodi, CA	60	0.63	1.15	1.15
Syracuse, NY	31	0.32	0.58	0.52
Tampa--St. Petersburg--Clearwater, FL	402	0.97	0.62	0.64
Toledo, OH	37	0.42	0.77	0.87
Tucson, AZ	64	0.44	0.80	0.63
Tulsa, OK	111	0.83	1.51	1.47
Honolulu (Urban), HI	48	0.33	0.59	0.47
Virginia Beach--Norfolk--Newport News, VA--NC	242	0.99	0.76	0.97
Washington--Arlington--Alexandria, DC--VA--MD--WV	1,105	1.34	1.19	0.90
Wichita, KS	19	0.21	0.39	0.45
Winston-Salem, NC	54	0.60	1.08	1.11
Worcester, MA--CT	33	0.24	0.44	0.37
Youngstown--Warren--Boardman, OH--PA	20	0.24	0.44	0.55

Supplement Table 6c. Rates of new diagnoses among men who have sex with men and who do not have an HIV diagnosis, 104 US metropolitan statistical areas, 2013

		Rate per 100 MSM without an HIV diagnosis		
**Metropolitan Statistical Area**	**n**	**Method 1**	**Method 2**	**Method 3**
Akron, OH	36	0.37	0.70	0.63
Albany--Schenectady--Troy, NY	33	0.27	0.52	0.38
Albuquerque, NM	47	0.39	0.75	0.56
Allentown--Bethlehem--Easton, PA--NJ	30	0.26	0.48	0.49
Atlanta--Sandy Springs--Roswell, GA	1,393	2.41	2.08	1.62
Augusta--Richmond County, GA--SC	89	1.25	2.51	3.91
Austin--Round Rock, TX	256	1.13	0.74	0.68
Bakersfield, CA	72	0.64	1.21	1.26
Baltimore--Columbia--Towson, MD	515	1.54	1.30	1.30
Baton Rouge, LA	155	1.55	3.22	4.14
Birmingham--Hoover, AL	130	0.95	0.64	0.92
Boise City, ID	13	0.15	0.28	0.35
Boston--Cambridge--Newton, MA--NH	423	0.69	0.61	0.49

Bridgeport--Stamford--Norwalk, CT	50	0.41	0.78	0.68
Buffalo--Cheektowaga--Niagara Falls, NY	80	0.51	0.31	0.39
Cape Coral--Fort Myers, FL	72	0.79	1.52	1.01
Charleston--North Charleston, SC	84	0.95	1.92	1.81
Charlotte--Concord--Gastonia, NC--SC	285	1.02	0.76	0.89
Chattanooga, TN--GA	35	0.50	0.97	1.02
Chicago--Naperville--Elgin, IL--IN--WI	1,361	1.17	0.80	0.87
Cincinnati, OH--KY--IN	168	0.60	0.47	0.57
Cleveland--Elyria, OH	218	0.82	0.55	0.68
Colorado Springs, CO	18	0.20	0.38	0.40
Columbia, SC	99	1.07	2.30	1.96
Columbus, OH	229	0.95	0.63	0.62
Dallas--Fort Worth--Arlington, TX	1,019	1.34	0.80	0.85
Dayton, OH	61	0.58	1.12	1.02
Deltona--Daytona Beach--Ormond Beach, FL	55	0.66	1.28	1.09
Denver--Aurora--Lakewood, CO	198	0.63	0.52	0.40
Des Moines--West Des Moines, IA	28	0.36	0.68	0.62
Detroit--Warren--Dearborn, MI	390	0.71	0.53	0.68
Durham--Chapel Hill, NC	70	1.08	2.21	1.34
El Paso, TX	96	1.01	2.04	3.11
Fresno, CA	88	0.75	1.46	1.35
Grand Rapids--Wyoming, MI	31	0.23	0.16	0.19
Greensboro--High Point, NC	75	0.83	1.67	1.58
Greenville--Anderson--Mauldin, SC	80	0.72	1.40	1.69
Harrisburg--Carlisle, PA	30	0.40	0.76	0.57
Hartford--West Hartford--East Hartford, CT	73	0.44	0.28	0.35
Houston--The Woodlands--Sugar Land, TX	1,013	1.42	0.89	1.11
Indianapolis--Carmel--Anderson, IN	175	0.74	0.53	0.58
Jackson, MS	106	1.72	3.93	4.05
Jacksonville, FL	210	1.23	0.80	1.07
Kansas City, MO--KS	154	0.61	0.48	0.52
Knoxville, TN	49	0.42	0.80	0.92
Lakeland--Winter Haven, FL	49	0.60	1.14	0.99
Lancaster, PA	27	0.38	0.70	0.58
Las Vegas--Henderson--Paradise, NV	292	1.17	0.63	0.69
Little Rock--North Little Rock--Conway, AR	124	1.41	2.87	2.87
Los Angeles--Long Beach--Anaheim, CA	1,938	1.31	0.68	0.71
Louisville--Jefferson County, KY--IN	136	0.85	0.57	0.63
Madison, WI	27	0.31	0.58	0.44
McAllen--Edinburg--Mission, TX	66	0.73	1.39	1.88
Memphis, TN--MS--AR	257	1.80	1.09	1.43
Miami--Fort Lauderdale--West Palm Beach, FL	1,592	2.67	1.83	1.35
Milwaukee--Waukesha--West Allis, WI	98	0.48	0.33	0.40

Minneapolis--St. Paul--Bloomington, MN--WI	177	0.40	0.28	0.26
Modesto, CA	15	0.22	0.42	0.59
Nashville--Davidson--Murfreesboro--Franklin, TN	181	0.85	0.65	0.70
New Haven--Milford, CT	56	0.48	0.93	0.83
New Orleans--Metairie, LA	356	2.67	2.09	2.08
New York--Newark--Jersey City, NY--NJ--PA	3,007	1.34	0.91	0.89
North Port--Sarasota--Bradenton, FL	54	0.54	1.04	0.76
Ogden--Clearfield, UT	6	0.08	0.14	0.18
Oklahoma City, OK	155	0.94	0.64	0.80
Omaha--Council Bluffs, NE--IA	35	0.30	0.57	0.48
Orlando--Kissimmee--Sanford, FL	368	1.41	0.95	0.92
Oxnard--Thousand Oaks--Ventura, CA	53	0.47	0.89	0.79
Palm Bay--Melbourne--Titusville, FL	45	0.58	1.11	0.99
Philadelphia--Camden--Wilmington, PA--NJ--DE--MD	614	0.80	0.67	0.67
Phoenix--Mesa--Scottsdale, AZ	406	0.75	0.43	0.47
Pittsburgh, PA	109	0.33	0.23	0.29
Portland--South Portland, ME	12	0.17	0.31	0.18
Portland--Vancouver--Hillsboro, OR--WA	139	0.46	0.36	0.28
Providence--Warwick, RI--MA	94	0.42	0.33	0.32
Provo--Orem, UT	3	0.04	0.08	0.14
Raleigh, NC	141	0.98	0.59	0.73
Richmond, VA	123	0.81	0.71	0.81
Riverside--San Bernardino--Ontario, CA	370	0.69	0.48	0.41
Rochester, NY	67	0.46	0.30	0.36
Sacramento--Roseville--Arden-Arcade, CA	149	0.52	0.34	0.35
St. Louis, MO--IL	233	0.66	0.61	0.69
Salt Lake City, UT	45	0.32	0.18	0.20
San Antonio--New Braunfels, TX	365	1.36	0.82	1.06
San Diego--Carlsbad--San Marcos, CA	425	1.12	0.59	0.59
San Francisco--Oakland--Hayward, CA	643	1.31	0.85	0.50
San Jose--Sunnyvale--Santa Clara, CA	109	0.43	0.24	0.31
Scranton--Wilkes-Barre--Hazelton, PA	11	0.13	0.25	0.30
Seattle--Tacoma--Bellevue, WA	245	0.53	0.36	0.32
Spokane--Spokane Valley, WA	11	0.15	0.27	0.23
Springfield, MA	41	0.49	0.93	0.86
Stockton--Lodi, CA	60	0.67	1.27	1.28
Syracuse, NY	31	0.34	0.64	0.56
Tampa--St. Petersburg--Clearwater, FL	402	1.12	0.67	0.70
Toledo, OH	37	0.45	0.86	0.99
Tucson, AZ	64	0.49	0.95	0.72
Tulsa, OK	111	0.90	1.75	1.70
Honolulu (Urban), HI	48	0.36	0.69	0.53
Virginia Beach--Norfolk--Newport News, VA--NC	242	1.15	0.85	1.13

Washington--Arlington--Alexandria, DC--VA--MD--WV	1,105	1.66	1.44	1.03
Wichita, KS	19	0.23	0.43	0.51
Winston-Salem, NC	54	0.65	1.28	1.33
Worcester, MA--CT	33	0.25	0.48	0.39
Youngstown--Warren--Boardman, OH--PA	20	0.25	0.47	0.60

Interpretation

In general, results were consistent between the three denominator methods, particularly at the state level. The single national MSM average Model 1 yielding the most inconsistent results, likely owing to having the most naïve assumptions. Several notable departures between the urbanicity-based model 2 and the urbanicity and ACS-based model 3 included San Francisco and DC. The former model yielded lower MSM denominators and implausibly high levels of HIV prevalence, likely due to a failure to detect these cities' unusually high densities of MSM, relative to their urbanicity level. Model 3 adjusted these estimates with a city-specific male-male cohabitation rate, yielding more plausible results. Conversely, at the MSA and county levels, some counties with extremely high prevalence per Method 3 were places with an above-average (for their level of urbanization) percent of the county population who were incarcerated. Persons who are diagnosed with HIV infection while incarcerated are counted as residing in the county of their incarceration, but incarcerated persons may not be included in the ACS information used in the denominators. Using the urbanicity-based Model 2, these prevalence values were reduced to more realistic but still high levels.

References

1. Grey JA, Bernstein KT, Sullivan PS, Purcell DW, Chesson HW, Gift TL, and Rosenberg ES. *Estimating the population sizes of men who have sex with men (MSM) in US states and counties using data from the American Community Survey.* JMIR Public Health and Surveillance, (in-press).

2. Oster AM, Sternberg M, Lansky A, Broz D, Wejnert C, and Paz-Bailey G. *Population Size Estimates for Men who Have Sex with Men and Persons who Inject Drugs.* J Urban Health, 2015.

3. Purcell DW, Johnson CH, Lansky A, Prejean J, Stein R, Denning P, Gau Z, Weinstock H, Su J, and Crepaz N. *Estimating the population size of men who have sex with men in the United States to obtain HIV and syphilis rates.* Open AIDS J, 2012. 6: p. 98-107.

4. Lieb S, Thompson DR, Misra S, Gates GJ, Duffus WA, Fallon SJ, Liberti TM, Foust EM, Malow RM, and Team SACMP. *Estimating Populations of Men Who Have Sex with Men in the Southern United States.* Journal of Urban Health-Bulletin of the New York Academy of Medicine, 2009. 86(6): p. 887-901.

5. Hall HI, An Q, Tang T, Song R, Chen M, Green T, Kang J, Centers for Disease C, and Prevention. *Prevalence of Diagnosed and Undiagnosed HIV Infection--United States, 2008-2012.* MMWR Morb Mortal Wkly Rep, 2015. 64(24): p. 657-62.
